# NURSING ASSISTANTS’ OPINIONS ON CREATING THE PERFECT DINING EXPERIENCE FOR RESIDENTS WITH DEMENTIA

**DOI:** 10.1093/geroni/igz038.1876

**Published:** 2019-11-08

**Authors:** Christine Ferguson, Joy W Douglas, Seung Eun Jung, Hyunjin Noh, Amy Ellis

**Affiliations:** 1 University of Alabama, Tuscaloosa, Alabama, United States; 2 The University of Alabama School of Social Work, Tuscaloosa, Alabama, United States

## Abstract

Feeding residents with dementia in long-term care settings can be challenging, partly related to environmental factors. Certified Nursing Assistants (CNAs) are primarily responsible for feeding residents with dementia who need assistance. Given that older adults with dementia have an increased risk of developing malnutrition, there is a need to develop standards in place for constructing an ideal dining environment to optimize residents’ dietary intake. This qualitative study was conducted to explore CNA’s perspectives of how dining areas could be enhanced to improve food intake of residents with dementia. Nine focus groups were conducted with a total of 53 CNAs who had at least one year of experience feeding residents. Focus groups were audio recorded and transcribed verbatim. Data were analyzed using directed content analysis guided by the Social Ecological Model. CNAs reported that distractions can significantly inhibit residents’ food intake; therefore, limiting distractions such as noise and crowding is important. CNAs also reported the benefit of playing music in the dining area depended on the individual resident. Additionally, CNAs emphasized the importance of offering a variety of appetizing menu choices tailored to residents’ preferences. CNAs have firsthand experience with residents with dementia and can provide valuable insights. Long-term care administration should consider interdisciplinary support to improve the mealtime experience of residents with dementia in an effort to enhance their dietary intake. In particular, providing a variety of menu choices in a well-lit, calm, spacious, and homelike dining environment can be beneficial.

